# Assessing Public Health Capacity for Infectious Disease Modeling: A Qualitative Study of State and Local Agencies

**DOI:** 10.3390/ijerph22081301

**Published:** 2025-08-20

**Authors:** Skyler J. Crouch, Katie S. Allen, Delaney Thornton, Joel Hartsell, Elizabeth H. Weybright, Julia E. Szymczak, Kimberley I. Shoaf

**Affiliations:** 1 Division of Public Health, Department of Family and Preventive Medicine, University of Utah, Salt Lake City, UT 84108, USA; kimberley.shoaf@utah.edu; 2 Epi-Vant, Salt Lake City, UT 84092, USA; katieallen@epi-vant.com (K.S.A.); joelhartsell@epi-vant.com (J.H.); 3 Division of Epidemiology, Department of Internal Medicine, Spencer Fox Eccles School of Medicine, University of Utah, Salt Lake City, UT 84112, USA; delaney.thornton@hsc.utah.edu (D.T.); julia.szymczak@hsc.utah.edu (J.E.S.); 4 Department of Human Development, Washington State University, Pullman, WA 99164, USA; elizabeth.weybright@wsu.edu

**Keywords:** infectious disease modeling, public health capacity, local health agencies

## Abstract

Infectious disease modeling and forecasting tools are crucial for outbreak management. However, variability exists in the capacity of state and local health departments to effectively utilize these tools, influenced by factors such as infrastructure, funding, staff capacity, and data access. This study aims to identify the current priorities, needs, and capacities of state and local public health departments regarding infectious disease modeling and forecasting tools. Key informant interviews were conducted with epidemiologists, informaticists, and leadership across state and local health departments from Montana, Utah, and Washington. Thematic coding and axial coding were used for thematic analysis. Three themes emerged: (1) models and tools must be adaptable based on the jurisdiction type (rural, urban, state); (2) building trust in models and tools is an important precursor to adoption; and (3) there are concerns about the availability and quality of data. This study highlights the need for adaptable modeling tools that are tailored to specific public health jurisdictions. Building trust in modeling and forecasting tools and addressing data quality issues are essential for successful tool implementation and adoption across diverse public health settings.

## 1. Introduction

Modeling and forecasting tools have increasingly become essential for managing infectious disease outbreaks [1]. These tools predict pathogen transmissibility, inform policy decisions, assess the effectiveness of control and prevention measures, and allocate essential resources during public health crises [1,2]. Accurate disease modeling has been accentuated by global challenges such as climate change, which influences disease vectors, and increased globalization, which facilitates the rapid spread of pathogens [2]. Timely access to reliable data is critical in these models, as outdated or incomplete information can lead to misinformed public health responses [3]. This need was particularly evident during the COVID-19 pandemic when real-time data informed containment strategies and protected vulnerable populations [4]. Beyond infectious diseases, modeling has played a key role in addressing chronic disease prevention and substance abuse, underscoring its growing role in improving population health outcomes [5].

Public health agencies have utilized models and forecasting tools in various ways during past outbreaks [5,6]. For instance, during the 2009 H1N1 influenza pandemic, the World Health Organization used models to coordinate outbreak response units and inform global vaccination strategies [5,6,7,8]. In the 2014–2016 West African Ebola outbreak, models estimated outbreak control parameters and informed the design of vaccination trials [5,7]. Infectious disease models have guided the “test-and-treat” strategy to prevent HIV transmission [5,8]. Effectiveness in these cases relied on models that were accurate, adaptable, and aligned with the resource capacities of each implementing agency, enabling timely interventions.

Despite these successes, the capacity for effectively using modeling and forecasting tools is uneven across the public health landscape [9,10]. While state health departments (SHDs) often have broader resources, infrastructure, and data access than local health departments (LHDs), LHDs operate closer to their communities, often with more limited budgets and staff [9,10]. Many local and state health departments lack the necessary infrastructure, funding, personnel, and experience to apply advanced infectious disease modeling techniques [5,10]. Conversely, model developers at academic institutions have the technical expertise to create complex models but may not understand the needs and capacities of public health agencies. These structural differences and varying priorities and resources highlight the differences in capacity for using models and forecasting within the public health context [9,10]. For models to be effective, they must be designed with varying levels of technical expertise, resource availability, and specific public health needs in mind [5].

Despite the growing importance of modeling tools in public health, much of the existing research focuses on the models themselves or on healthcare systems capacities to implement them [2,7,8,11]. Far less attention has been paid to the capacity of public health agencies, particularly at the state and local levels, to adopt and use these tools effectively. This study addresses that gap by providing qualitative insights into the jurisdiction-specific needs, priorities, and constraints that shape public health engagement with infectious disease models.

To ensure public health infectious disease models are effective and functional for state and local health departments, it is essential for model developers to first understand the specific needs of public health agencies and how they vary by jurisdiction. The objective of this study was to identify the current priorities, needs, and capacities of state and local public health departments for infectious disease modeling and prediction tools within the U.S. states of Montana, Washington, and Utah. The results will guide the development of infectious disease models by the Mountain West Center for Forecasting and Surveillance of Infectious Threats and Epidemics (ForeSITE). ForeSITE is one of thirteen centers funded by the Centers for Disease Control and Prevention (CDC) Center for Forecasting and Outbreak Analytics (CFA) to establish a national outbreak response network and create tools to support decision-making in public health emergencies [12]. This analysis reports the results of the qualitative work and offers recommendations for how model developers can approach public health modeling and forecasting.

## 2. Materials and Methods

### 2.1. Study Design

We conducted a qualitative formative evaluation, utilizing in-depth key informant interviews to understand how infectious disease models have been used by SHDs and LHDs, and to assess public health needs for future tool development. A semi-structured interview guide was developed to facilitate the gathering of the perspectives of potential users.

### 2.2. Study Population

To be eligible for the study, participants had to be working for an SHD or LHD within the states of Montana, Washington, or Utah at the time of the interviews and needed to have some experience with using an infectious disease model or forecast to inform public health decision-making at the state or local level. Purposive sampling was used to identify participants across state and local health departments with a focus on individuals working as informaticists, epidemiologists, and in leadership positions. Initial contact was conducted via email and included a study description and a request to identify additional participants within their agencies, with follow-up emails sent approximately two weeks later.

### 2.3. Data Collection

An interview guide was developed with input from public health and modeling experts and explored topics such as experience with infectious disease models and tools; barriers to their use; data collection and utilization for infectious disease surveillance and interventions; infectious diseases for which modeling tools are most beneficial; and confidence in model outputs (see Supplementary Materials). While the study focused on general experiences and needs related to infectious disease models and forecasts, it did not aim to evaluate specific tools or track which tools were used by individual agencies. Participants were allowed to participate in an interview individually or as a group, and the interviews included a primary interviewer and at least one note-taker (KSA, SJC, DT). The interviews were semi-structured, conducted virtually on Zoom, and lasted between 40 to 60 min. Each interview was audio and video recorded, transcribed verbatim, and reviewed for accuracy by a member of the study team (SJC) before being imported to Dedoose (version 9.0.17) for analysis [13].

### 2.4. Data Analysis

The codebook for thematic analysis was developed deductively based on the interview guide and included themes such as organizational capacity; data sources and availability; current infectious disease processes, priorities, and interventions; experience with relevant tools; technical infrastructure; and resource availability (see Supplementary Materials). Two team members (SJC, DT) completed the thematic coding individually in Dedoose (version 9.0.17) [13], and disagreements were resolved via consensus and arbitration (KSA). The coding team met routinely to adjudicate coding. Axial coding for thematic analysis was completed inductively (KSA) with consensus review by all authors.

### 2.5. Ethical Considerations

The University of Utah Institutional Review Board determined this evaluation to be exempt (IRB_00175977). All participants provided informed consent and agreed to audio and video recording. To maintain confidentiality, participants were identified in transcripts using a generic label followed by a sequential number (e.g., “participant 1”).

## 3. Results

We conducted 16 interviews with 23 participants from 3 SHDs and 6 LHDs in Montana, Utah, and Washington. No notable differences were observed across states in terms of participant responses, and, as such, data from all three states were combined for analysis. SHDs consistently reported having an informatics department, modeling experience, and interactions with tribes and federal entities. LHDs reported a range of experiences, with half having an informatics department, some experience with models, and interactions with tribes and federal entities. The participants represented various professional roles, often holding more than one position, including 14 epidemiologists, 11 from leadership roles, 8 informaticians, and 1 public health nurse (Table 1). Participants identified vaccine-preventable diseases, respiratory viruses, syphilis, measles, enteric diseases, and COVID-19 as the most commonly prioritized conditions for model use.

The primary themes identified during thematic analysis were: (1) the variation observed between state and local health departments requires adapting the approach to the model or tool implementation based on the setting; (2) the trust in models and tools by the user is an important precursor to adoption and should be considered in implementation planning; and (3) concerns about the availability and quality of data need to be addressed when planning for model or tool implementation. We present these identified themes with relevant quotes below.

### 3.1. Theme 1: The Model or Tool Must Be Adaptable Based on the Jurisdiction Type (Rural, Urban, State)

Differences in priorities and capacities were evident between LHDs and SHDs. LHDs have an emphasis on intervention via case management, whereas SHDs have an emphasis on surveillance. Local health departments, particularly rural and smaller LHDs, lack sufficient technical infrastructure to host data and the necessary computing power internally. Instead, they rely on surveillance data collected by and/or hosted at the state level. This dynamic results in LHDs attempting to integrate models and tools that do not meet their priorities, and SHDs using tools that limit their work or reduce their capacity, highlighting the importance of tools that can meet the unique priorities and capacities of different health departments.

*“I know some of our jurisdictions would probably utilize such tools depending on the level of expertise required. Other ones may not. I mean, we have counties here which are smaller than our Department of Health in terms of population, and their local public health jurisdiction may essentially be the equivalent of half an FTE of a public health nurse.”*

*—State health department, informaticist*

Limited funding and staff resources emerged as additional challenges in the development and maintenance of models. Participants at both state and local levels expressed an interest in adopting new technologies but lacked the capacity to learn the required new skills due to resource constraints. As such, internal and public-facing dashboards emerged during the COVID-19 pandemic as the primary tools utilized by both LHDs and SHDs. These dashboards typically display simple incidence rates and rely on Application Programming Interface (API) to connect the dashboards with data sources, bringing the technology within reach of many local and state health departments. However, more complex models and tools are in limited use due to the previously mentioned limitation of resources.

*“One of the challenges I see is that I don’t want tools that reduce our capacity. If tools try and do too much and don’t allow you enough access into them, then they can effectively reduce your capacity rather than increase it. That’s one challenge, I think, that all tool developers face.”*

*—State health department, informaticist*

*“Ultimately, it comes down to staffing, time, money. Public health never has the resources that we want or need. And it’s not for lack of expertise, which is something that frustrates me, as people are like, ‘Oh, government workers don’t know how to build models.’ And that’s not true. We just have so many other competing priorities.”*

*—Local health department, epidemiologist*

### 3.2. Theme 2: Building Trust in Models and Tools Is an Important Precursor to Adoption

Some participants mentioned distrust in models, primarily related to novelty, unfamiliarity, distrust in underlying data, and the proliferation of available models. Some models that were used during the COVID-19 pandemic were deemed unusable due to inaccurate and/or inconsistent predictions. Some of these inaccuracies were attributed to low case counts, which cause known issues related to model accuracy at the granularity required to be useful to the LHDs. For example, models utilize data aggregated across multiple jurisdictions to ensure high enough case counts for statistical accuracy. In turn, the model is no longer applicable to the local public health agency. However, disaggregated data with low counts produces predictions that are not trustworthy.

Trust was further broken during the pandemic as models primarily used for chronic conditions were quickly and poorly adapted for use with infectious diseases. LHDs attempted to leverage these tools, which were ultimately unhelpful or inaccurate. This has created an overall skepticism related to modeling and forecasting tools. These perceptions have continued into the post-pandemic landscape, impacting the users’ trust in models. Efforts to showcase accuracy and applicability must be undertaken to regain trust and facilitate the implementation of models in the future.

There is also concern about the usefulness of models outside of very specific needs. For example, a model may be useful for identifying the true prevalence of a condition, but the implementers may deem the model unusable if it does not meet their need to communicate the information in a digestible format for the public and providers. Additionally, scenario modeling has the potential to help LHDs and SHDs; however, it should only include elements that can be intervened on (i.e., masking should not be included as a modeling element if masking policies are not allowed within the jurisdiction). Lastly, participants from rural LHDs expressed concerns about model usefulness in areas with low population density, especially if the jurisdiction includes a large rural land area with only one or two population centers.

*“I don’t know that we’re in a state where policymakers and public health would trust model projections or forecasts that much. I actually think we’re in a state where they shouldn’t trust them that much … because, most often, of unfamiliarity. That’s the primary thing. Why they shouldn’t [trust models] is because they’re still not very good, at least on the COVID forecasting side.”*

*—State health department, informaticist*

*“We’ve talked about the shortcomings of models in small rural areas. We don’t have the volume. Our studies don’t have the power of making the models especially useful.”*

*—Local health department, epidemiologist*

### 3.3. Theme 3: There Are Concerns About the Availability and Quality of Data

Data governance was a frequently raised concern for the usefulness and accuracy of modeling and forecasting tools. This applied to both inter-agency data sharing as well as cross-jurisdictional sharing. State and local health departments rely on a variety of data sources for dashboards, surveillance, and interventions. Primary data sources include laboratory and case reporting mandated by jurisdictions’ reporting rules. However, LHDs rely heavily on case investigation and contact tracing notes for their intervention activities. To some extent, state and county repositories (i.e., vaccination databases and vital records) are also utilized. However, these data sources have varying levels of completeness. In some instances, electronic records rely heavily on the manual review of clinicians’ notes and billing codes, which lack standardization and may vary from one health system to another. Some variables, such as race and ethnicity, addresses, and phone numbers, were mentioned as having high levels of incompleteness, resulting in manual efforts using traditional contact tracing methods. Additionally, some rural LHD facilities are not yet fully operationalized for electronic reporting, removing electronic records as a data source entirely. Jurisdictions in rural areas indicated minimal electronic case reporting, often with their health systems still relying on fax machines to receive case reports.

*“Depending on the reporting lab or facility, we definitely have some that are much more thorough in what they report. … But you are going to always be missing an address or a phone number. … You’re going to be missing some of those details that you need right away to start that investigation process.”*

*—Local health department, epidemiologist*

*“Another issue of poor data quality could be found at vital records because the record is put in by the physician filling it out. And so there’s no standardization of their immediate cause of death. For one person, it could be a myocardial infarction. The other person could write ‘heart attack.’ We both know that those are the same thing, but when you’re trying to code it or you’re trying to look at it, it’s impossible.”*

*—Local health department, epidemiologist*

*“The way we get our notifiable conditions reported to us is essentially through a digital fax portal. Prior to the pandemic, we had the old-school fax machine that would just spit out paper. … Then we moved to that digital fax portal to be able to handle that data flow. But that’s still a little clunky and outdated. We’re not quite up to speed with the electric lab reporting. For a small county, that’s something that we’d like to have, but it may not be financially feasible for us to do it.”*

*—Local health department, leadership*

Data sharing capability is another element that hampers data availability. Data-sharing procedures vary across jurisdictions, even interdepartmentally at the State level. LHDs reported difficulty in acquiring data from the SHDs for their jurisdictions, although it should be noted that SHDs reported that the data is readily available to LHDs if the proper procedures are followed. Participants mentioned the desire to access data from neighboring states, counties, tribal lands, and health districts to combat multi-jurisdictional outbreaks, but restrictive data-sharing procedures present major challenges that are often insurmountable. Addressing data governance issues could alleviate these challenges and ensure models are usable across jurisdictions.

*“What I really wanted to be captured in this interview was our struggles with data sharing and the reality of that across ForeSITE or whatever. You’re going to have states that manage this really easily and states that are like us. It’s a hell of a day to try and get that shared.”*

*—State health department, informaticist*

*“Data sharing keeps me up at night every single day, because, especially in this partnership, we are really struggling. … We interpret law very conservatively. Sharing data below state level is very difficult and often not going to happen. As we’re thinking through this partnership, I think analysis and models can look very different at a state level versus how they might look at a county level, or a local health department level, or zip code level.”*

*—State health department, informaticist*

*“Technically, we have access [to county line-level data], but we have to request it from the state, and that’s a process. It’s many iterations of back and forth of, ‘Can we alter the query this way?’, ‘I’m finding these errors,’ etc.”*

*—Local health department, informaticist*

Lastly, due to the low population sizes, many rural and smaller health departments lack sufficient case counts of infectious diseases to ensure patient privacy when case counts are fewer than 11. This necessitates the removal of potentially identifiable information like age, race and ethnicity, and sometimes gender. However, this also implies that rural and smaller LHDs may be deliberately missing variables that could prove useful in models and tools.

*“One of the data sharing arguments is that if your data is less than 11, you can’t share it with outside people. It becomes very challenging because I feel like I’m sitting on the data and can’t share it with anyone.”*

*—Local health department, epidemiologist*

*“We have a dashboard created specifically for our county that we use. But with it being a small county and a small population, sometimes those numbers may not be as statistically significant as the larger data dashboard.”*

*—Local health department, leadership*

## 4. Discussion

The objective of our study was to identify the current priorities, needs, and capacities of state and local public health departments for infectious disease modeling and prediction tools within Montana, Washington, and Utah. Our findings highlight the need for adaptable modeling and forecasting tools that accommodate the diverse capabilities and priorities of state and local health departments. Specifically, we observed significant disparities in resources, technical infrastructure, data availability, and expertise in disease modeling between rural and urban health departments and between local and state health departments. These disparities are not unique to our study and have been documented in other public health contexts, such as national surveys and reports conducted by the National Association of County and City Health Officials (NACCHO) [5,14,15,16,17]. However, there are relatively few peer-reviewed articles post-COVID-19 that specifically examine the capacity of public health agencies to incorporate infectious disease models into practice [18]. Our work aims to bridge this gap by providing qualitative evidence from multiple jurisdictions.

While SHDs often have the capacity to handle advanced modeling, smaller LHDs face significant challenges, including a lack of informatics departments, staff who fill multiple roles, and reliance on the SHD for data management and surveillance [10,17,18,19,20,21]. The resource gap, particularly in technical infrastructure and personnel, places smaller LHDs at a distinct disadvantage when responding to outbreaks [21]. For instance, many rural LHDs lack access to the computational tools and server infrastructure to host complex models, relying instead on state agencies for data storage and processing [10]. This reliance limits their ability to generate real-time insights, forcing them to adopt reactive rather than proactive strategies [21]. Furthermore, these infrastructure limitations are compounded by broader systemic constraints. According to a 2019 NACCHO report, most LHDs experienced stagnant or declining preparedness budgets and continued to rely heavily on state and local funding sources [21]. At the same time, the local public health workforce declined by approximately 16% between 2008 and 2019, with minimal growth in preparedness-specific roles such as epidemiologists and statisticians [21]. Capacity building and developing scalable tools could bridge this gap and ensure smaller health departments can access the necessary tools to manage outbreaks effectively.

One barrier to implementing models is a lack of trust in the models. Participants expressed concerns about the accuracy of models, often reporting experiencing inconsistent or unreliable predictions during the COVID-19 pandemic. This suggests that trust in modeling tools must be rebuilt with clear communication about the model’s limitations and strengths. However, building trust in models goes beyond improving transparency; it also requires local validation [3]. Models that are developed and tested using jurisdiction-specific data may be more likely to gain the confidence of public health professionals [3]. Regular updates based on local surveillance data, as well as the creation of user-friendly interfaces that can be effectively shared with the public and policymakers, can enhance the usability and credibility of models, as reported by Muscatello et al. (2017) [22]. Additionally, engaging local health departments in the design process ensures that models are aligned with the practice needs of these departments [3,22]. This participatory approach can mitigate skepticism and foster a sense of ownership over the tools being implemented.

Additionally, concerns about data quality and availability emerged as a critical issue. Inconsistent and incomplete data reduce the accuracy and usability of tools [11]. These data quality issues arise for a variety of reasons. First, there is a lack of harmonization in data collection across data sources, including at the health system level. This presents issues for the incorporation of data into analysis, limiting comparability. Second, though health IT standards have advanced significantly over the past two decades, there remain variability in content caused by clinical workflows as well as technical limitations. Dixon et al. (2023) emphasized that the fragmented nature of the public health information systems and reliance on manual data entry during the COVID-19 pandemic hindered timely access to high-quality data [23]. They advocate for investments in analytics capabilities and interoperable infrastructure to support forecasting and public health decision-making [23]. While technical modeling capabilities continue to advance, our findings suggest that data governance and sharing protocols may pose more significant barriers to effective model implementation than the models themselves. Future modeling efforts may also benefit from approaches that are robust to incomplete or uncertain data, such as Bayesian regression or multivariate imputation by chained equations (MICE), which can help mitigate the impact of data limitations [24].

Rural jurisdictions face particular challenges due to lower population densities, including insufficient case counts to protect patient privacy, which limits the ability to use the data in modeling [3,25]. The challenge of data sharing, particularly across jurisdictions, continues to be a significant barrier to effective disease modeling and outbreak response [16]. Current policies often restrict the flow of information between states, local health departments, and tribal and federal entities, resulting in fragmented datasets that limit the accuracy of models [16]. These findings are consistent with van Panhuis et al. (2014), who identified twenty distinct barriers to public health data sharing, including technical limitations, restrictive policies, and concerns about privacy in small populations [26]. Establishing regional or national data-sharing frameworks, such as a health information exchange, which facilitate the real-time exchange of information could significantly improve the ability of health departments to respond to public health emergencies [16,23]. These frameworks should also consider robust privacy protections to address concerns about sensitive data in smaller jurisdictions, where limited case counts increase the risk of identifying individuals. Addressing these challenges will require technical solutions (such as ensuring data standardization) and policy interventions supporting more effective data sharing.

Our findings reveal several actionable recommendations that could improve the usability and effectiveness of modeling tools for diverse public health departments. As tool developers move forward with creating infectious disease models and forecasts for public health, we recommend that the following guidelines be taken into consideration:

First, distinct views are needed across local and state health departments. Models need the ability to be customized to jurisdiction-level needs or concerns, including elements that can be intervened upon at the level at which they are being used.

Second, fully understanding the reasoning behind model creation is key to trust and facilitating interpretation. Incorporate partners in testing or pilot activities to facilitate buy-in and trust.

Third, developers should ensure the data required for the model is available at all levels and that the data is sufficient for the use case (i.e., the data needs to have high levels of completeness and quality).

Lastly, governance needs to be fully considered prior to model development. Explore whether implementation at the state level removes some of the governance considerations.

## 5. Conclusions

As modeling becomes an increasingly indispensable tool in public health, understanding the diverse needs of health departments is crucial. This study aimed to explore the current priorities, needs, and challenges public health departments face in adopting infectious disease modeling tools. Our findings underscore several critical needs: first, there are diverse capacities and priorities of LHDs and SHDs, along with differences between rural and urban settings; second, there is distrust in models across all levels of jurisdiction; and lastly, data quality and data governance issues are of concern. To bridge these gaps, we recommend several key actions. Modeling tools should be customizable to meet jurisdiction-specific needs, data governance and availability issues must be prioritized, and public health stakeholders should be involved in model testing and implementation. Finally, ongoing research and development are necessary to support public health departments in managing future public health emergencies. Ensuring accessible, high-quality data and stakeholder-engaged model development can enable a more resilient public health system that is equipped to respond to infectious disease threats in an increasingly interconnected world.

## Figures and Tables

**Table 1:** Count of interviewed roles by jurisdiction type.

Role	State Health Department	Local Health Department	Total Count
Epidemiologist	5	9	14
Informaticist	4	4	8
Leadership	4	7	11
Public Health Nurse	0	1	1

## Data Availability

The data presented in this study are available on request from the corresponding author due to privacy reasons.

## Supplementary Materials

The following supporting information can be downloaded at: https://www.mdpi.com/article/10.3390/ijerph22081301/s1, Figure S1: Focus Group Semi-Structured Interview Questions; Table S1: Thematic Analysis Codebook.
