# Supplementary material for: Assessing Public Health Capacity for Infectious Disease Modeling: A Qualitative Study of State and Local Agencies
Source: Int J Environ Res Public Health. 2025 Aug 20;22(8):1301. doi: 10.3390/ijerph22081301 (PMC12386532; doi:10.3390/ijerph22081301)
Supplement: Supplementary file 1 [file ijerph-22-01301-s001.zip › ijerph-3708031_Supplement1.pdf]

Focus Group Semi-Structured Interview Questions

Version 1.3, last updated: 1/23/2024

Thank you for taking the time to meet with us today. As you know, we have been tasked with understanding the needs of the health departments and healthcare systems as it relates to the ForeSITE project. To accomplish this, we are undertaking a series of interviews with public health departments and other stakeholders. Findings from these interviews will be aggregated, individual and organizational identifiers will be removed. With your permission, I'd like to record today's interview for note-taking purposes and to ensure accuracy.

The ForeSITE project is a CDC funded program to provide data and tools that guide decisions to improve responses to emerging public health threats in the Mountain West and is part of a wider CDC initiative aiming to establish an outbreak response network. To make sure these programs are effective, we want to know how infectious disease modeling can support public health decision making. We're conducting a series of focus groups to discuss current activities and data, as well as what is missing, that would be helpful.

To set the stage, we'd like you to envision your activities around pandemic-potential, infectious diseases, including vaccine preventable or vector-borne diseases. For example, this could be the respiratory diseases you are currently battling but may also include conditions like tuberculosis, HIV, measles, or West Nile virus.

Which infectious diseases do you feel are most in need of models to support decision making?

- Do these vary by subgroup (for example, high school students, nursing home)?
- Which types of settings are the most critical and why? (For example, reporting from healthcare is strong but reporting from workplaces is less so.)
- Which types of transmission pathways are most critical and why? Are there specific populations you don't know about?
- Do these needs vary over a calendar year?
- What is it about these needs that make them the most critical?

Thinking about how you currently conduct surveillance activities:

- What data do you utilize to conduct surveillance?
- How do you access these data?
- What data elements are you missing that would be helpful to these activities? This could be gaps in your current surveillance systems, gaps in surveillance overall, or gaps in your ability to access collected data.

Thinking about how you currently conduct intervention activities (may need to take these questions by diseases defined in earlier question):

- What data do you need to address concerns in your community?
- Of the items needed, what data resources do you currently have access to?
- What data elements or information are you missing that would be helpful to these activities? Of those, which would be the highest priority and why?
    - Why is this data/information missing?

For the next series of questions, we'll be discussing disease forecasts or models. For this discussion, a model is defined as a process for identifying how an outbreak will unfold and how interventions will change that model. Forecasting refers to processes utilized when an outbreak is occurring to determine how that outbreak will go from this point forward. (Analogy: climate vs. weather)

Does your jurisdiction/organization use any infectious disease forecasts or models? These can be tools developed in your department, or you could have used an existing external resource.

If forecasts/modeling tools are utilized, walk me through how these models are being utilized.
Potential prompts:

- Can you tell me about your use of forecasting and modeling tools for infectious disease?
    - Which forecasting or modeling resources or tools do you currently leverage?
        - Which do you use the most?
        - Which do you find the most helpful and why?
    - Who/which groups in your organization currently use these models?
    - In your opinion, are there other members or programs who would gain value from the use of these models?
    - What do you wish those tools did that they currently do not?
        - Potential probes: different visualizations, ability to download data or graphics
    - Who provides and/or maintains those tools?
    - What, if any, barriers exist to utilizing these tools?
    - How are these tools used to guide decision making in your organization?
        - Probes: recommendations to decision makers, prioritize workflow, ration resources, etc.
    - Thinking back to a major public health event, for example COVID, when were these tools most useful? (E.g., outbreak response, resource allocation, etc.)
        - Walk me through how you used this tool. What worked? What didn't work? What would you like to see differently in the future?
        - What characteristics of these tools were most useful to your organization?
        - What characteristics were not useful or needed improved?
    - How has your use of tools changed in the past few years?
    - What are the major considerations when choosing if and how to use a tool?

If they are not using tools:

- What are the barriers or reasons for not utilizing modeling tools?
    - Are there groups within your organization that would benefit from these types of tools?
- Thinking back to a major public health event, for example COVID, do you think these types of tools would be beneficial to your decision-making processes?
    - Probes: recommendations, prioritize workflow, ration resources, etc.
    - What characteristics do you think would be most beneficial?
- When you are selecting the types of tools to be used for decision making, what are the major considerations?

At your organization, who is involved in the development and/or interpretation of models?

- What skillsets and resources do this person(s) have?

- Which additional skillsets and resources do you feel would be helpful?
- What tools would make the interpretation of these models easier?

How confident are you in the results of models that you have seen in the past?

  - Is there anything that would make you trust it more or less?

Is there anything additional we did not ask you about that you would like to share with us?

As this project progresses, what is the best way to contact you with follow-up questions?

Potential Adds
What were some of the presentation techniques that helped you understand complex information rapidly?
What kind of information do you need to communicate with others? (e.g., ppt decks with graphs? Executive decision memo?)
