# Supplementary material for: Assessing Public Health Capacity for Infectious Disease Modeling: A Qualitative Study of State and Local Agencies
Source: Int J Environ Res Public Health. 2025 Aug 20;22(8):1301. doi: 10.3390/ijerph22081301 (PMC12386532; doi:10.3390/ijerph22081301)
Supplement: Supplementary file 1 [file ijerph-22-01301-s001.zip › ijerph-3708031_Supplement2.pdf]

Supplementary Material – Thematic Analysis Codebook

Codes and subcodes	Definition
**Current Process**	Applied to the mention of current methods for conducting surveillance and intervention activities.
**Data Availability**	Represents any mention of what data the organization currently has access to or utilizes.
**Data Source**	Source of data used for tools.
**Need to review**	This code should be utilized if there is confusion/lack of clarity and the coder would like it elevated to be discussed by all coders.
**Organizational Capacity**	
Resource Availability	The availability of staff or other resources to facilitate the work.
Resource Skillset	The technical skills and abilities needed to facilitate the work.
**Population/Setting**	A priority population or setting for the organization.
**Public Health Role**	The self-identified role of the interviewee (select sub-code).
Leadership	
Epidemiologist	
Informatician	
Public Health Nurse	
**Technical Infrastructure**	The capabilities of the organization from a technology perspective. May include references to servers, how they currently access technology.
	The preference of the organization to host technology locally versus centralized at the State level.
**Tools and Experience**	Current tools or experiences with tools utilized for surveillance and interventions.
